# The Enduring Promise of Personalising Patient Preference Prediction

**DOI:** 10.1007/s12152-026-09635-7

**Published:** 2026-04-10

**Authors:** Brian D. Earp, Sebastian Porsdam Mann, Tessa van Veenendaal, Jemima Allen, Sabine Salloch, Karin Jongsma, Matthias Braun, Walter Sinnott-Armstrong, Julian Savulescu, David Wendler, Annette Rid

**Affiliations:** 1 Centre for Biomedical Ethics, Yong Loo Lin School of Medicine, National University of Singapore, Singapore, Singapore; 2 Uehiro Oxford Institute, University of Oxford, Oxford, United Kingdom; 3 Center for Advanced Studies in Bioscience Innovation Law, University of Copenhagen, Copenhagen, Denmark; 4 Institute for Ethics, History and Philosophy of Medicine, Hannover Medical School, Hanover, Germany; 5 Julius Center, University Medical Center Utrecht, Utrecht, Netherlands; 6 Department of (Social) Ethics, Faculty of Systematic Theology, University of Bonn, Bonn, Germany; 7 Department of Philosophy and the Kenan Institute for Ethics, Duke University, Durham, USA; 8 Clinical Center Department of Bioethics, National Institutes of Health, Bethesda, USA

**Keywords:** Personalized patient preference predictor, Autonomy, Substituted judgment, Advanced decision making

## Abstract

The challenge of making healthcare decisions for incapacitated patients continues to confront stakeholders worldwide. Annette Rid and David Wendler proposed a Patient Preference Predictor (P3) that uses population-level data to infer an incapacitated patient’s likely treatment choices, with the aim of aligning care with the values and preferences they held when last autonomous. Some objectors claimed this would fail to respect patients’ (former) autonomy because the basis for prediction would not be specific to the individual (e.g., based on data reflecting their own specific reasons for preferring one course of action over another). In response, we proposed a ‘Personalised Patient Preference Predictor’ (P4) that would harness the predictive capacities of personalised large language models (LLMs) fine-tuned on individual-level data of various kinds. The envisioned P4, if realized, would be akin to a ‘digital psychological twin’ or AI simulation of the patient that would encode their unique preferences and values to enable an individualised prediction of their likely treatment preferences. The P4, in turn, has been criticised on various grounds: philosophical, practical, and ethical. Here, we comprehensively evaluate the concerns of our critics based on all known published critiques as of the time of writing. While acknowledging the weight of some of these concerns, we argue that they do not entail that a P4 should not be developed. Rather, the concerns point to areas where thoughtful design choices, responsible regulation, and further philosophical reflection are needed to steer the proposal in a positive direction.

## Introduction

When a person loses the capacity to make their own healthcare decisions, alternative ways of deciding are needed. Frequently, surrogate decision-makers are involved. The substituted judgment standard holds that the surrogate(s) should strive to decide as the patient themselves would decide if they had capacity. This is often seen as a way of respecting the former autonomy of the patient: it anchors the decision in the preferences and values they held, or are reasonably believed to have held, when they were last autonomous.^1^ However, evidence suggests that human surrogates are often inaccurate in their predictions about how patients want to be treated in the event that they lose capacity [5]. Moreover, making substituted judgments is often very stressful for surrogates who may feel they have the (sole) responsibility to make a prediction, based on limited information, that can have life or death significance for a loved one [5–7].

These decisions are particularly challenging because the patient's best clinical interests are often unclear or contested. In many cases, there is no single medically superior option. Instead, the choice depends heavily on how the patient would weigh different risks, benefits, and outcomes based on their individual values and preferences. If there were a clear best treatment from a clinical standpoint, we could simply default to that. But when medical uncertainty exists or when trade-offs must be made between competing factors (such as longevity versus quality of life), accurately understanding what the patient would have wanted becomes crucial.

To address these challenges, Rid and Wendler [8, 9] proposed a Patient Preference Predictor (P3) that would use correlations between population-level hypothetical treatment preferences (as gathered through large scale surveys) and survey respondent demographic information, so as to predict how specific patients—when they were last competent—would want to be treated (i.e., based on their demographic features). However, critics argued that the use of group-demographic-based statistical associations to derive such predictions, even if accurate, would fail to truly respect patients by virtue of their former autonomy. Instead, they argued, such respect would require that predictions be based on individual-level factors such as the patient’s own endorsed preferences and values as these existed when the person was last autonomous.^2^

In response to these concerns, we recently proposed a 'Personalized Patient Preference Predictor' (P4) that, we argued, could address some of the claimed limitations of population-level prediction by using large language models (LLMs) fine-tuned on data produced by or otherwise appropriately related to (e.g., data describing) the individual patient [13]. This hypothetical digital system aims to predict treatment preferences in cases of decisional incapacity based on information specific to that person. Although a detailed prototype of our technical proposal has not yet been completed and formally tested (however, we are working on this), existing technologies and associated empirical findings make it plausible that a P4 could be developed to support surrogate decision-making for incapacitated persons [13]. Indeed, since our initial proposal, researchers have developed a preliminary P4-like prototype (albeit using somewhat different machine-learning methods), with apparently promising early results. As reported by the model’s developers in a recent *NEJM AI* report: “With a mean … accuracy of up to 70.6% (standard deviation ± 1.3%), our [prototype] performed on par with or better than typical estimates of [human] surrogate predictive accuracy in the literature” [14].

In our original contribution, we argued that the P4 may have certain advantages over other alternatives such as the previously proposed P3. For example, it might mitigate some of the above-described concerns about using (solely) population-level data to predict individual-level preferences. It could could also turn out to be more accurate than either population-level algorithms or typical human surrogates at correctly inferring the patient’s underlying preferences and values and, on that basis, predicting what the patient would choose if they had capacity. And, depending on how it was introduced and used in practice, it might help to ameliorate some of the intense stress faced by human surrogates, who may feel comforted by the knowledge that the P4’s prediction (however it is ultimately factored into the decision-making process) is based on information specific to their loved one.

Our article received fifteen responses in the *American Journal of Bioethics* (AJOB) and one other detailed response published separately in the *Journal of Medical Ethics* (JME) [1],^3^ the latter of which has, itself, received multiple commentaries [16–20] including one by us ​​[3]. Although we have briefly replied to the AJOB commentaries elsewhere [21, 22], in this paper, we will respond more thoroughly to the concerns raised by our critics, not only in AJOB, but across all known sources at the time of writing, including some of the respondents to the JME contribution and others who have published separate discussions in other journals.^4^ Thus, this paper represents our most up-to-date and comprehensive discussion of the ethical issues raised by the P4 to date. We are grateful to all the authors who have taken seriously our proposal and offered their considered replies. Already, their insights have been invaluable in refining our thinking and highlighting important opportunities for further research (see Box 1).

**Box 1 **Constructive feedback on the P4 proposal

**Table:** 

Some authors have provided constructive feedback that will help guide future specification of the P4 concept, or even help to inform its potential practical development and/or eventual application. For example, Ballantyne and Style [26] suggested an additional possible use of the P4: namely, that it could be used to ‘auto-fill’ advance directives for individuals who may, in the future, become patients requiring a substituted judgment, albeit before they lose decision-making capacity. In this way, the P4’s predictions could be approved and/or edited in advance by the individual (potentially with the involvement of their family as appropriate and desired). A similar concept in the form of a P4-Assisted Living Will was suggested by Milian and Bhattacharyya [20]. Li et al. [27] have highlighted the need to consider the P4 in non-Western cultures—particularly highlighting the linguistic and cultural adaptations that would be necessary for successful implementation. For example, Li et al. [27] argue that, in China, LLMs should be optimized through the incorporation of domain-specific knowledge that has been specifically adapted to the Chinese medical landscape.	

In addition to these positive contributions, there have also been some apparent misunderstandings about our proposal, and we will take this opportunity to correct them. We also respond to various objections that have been raised. We argue that while some of the concerns are undoubtedly valid, they do not entail that a P4 should not be developed. Rather, they highlight areas where thoughtful design choices, responsible regulation, and further philosophical reflection are needed to steer the proposal in a positive direction. We also agree with some of our respondents that robust empirical testing (i.e., of a P4 prototype and its descendants) is required to fully evaluate the proposal’s merits and drawbacks. Some of us are actively working on this.

Our paper is organized around four main areas of concern corresponding roughly to those identified previously in Earp et al. [21], albeit here, with more in-depth discussion, including responses to potential counter-arguments that we did not have space to address in our brief reply to the AJOB commentaries:

- **Technical concerns:** the P4 is technically undermotivated or infeasible [24, 28–32];
- **Data-related concerns:** developing the P4 raises data-related ethical concerns, notably around privacy risks and potential threats to authenticity if social media data were used for training [16, 20, 23, 26, 29, 33–39],
- **Justification given benefits/harms and alternatives:** the required accuracy threshold of the P4 to mitigate its potential harms and/or justify the resources necessary to build it, given alternative approaches, is too high [26, 28, 38–40]; and
- **Relationship-related concerns:** the potential damage to human relationships is prohibitive [16, 23, 37–39, 41, 42].

In what follows, we lay out the identified objections, respond to them in turn, and briefly outline some further thoughts in a final section before concluding.

## Technical Concerns (T)

Critics have raised concerns regarding the technical feasibility of the P4, which we have further divided into the following subcategories: **T1:** LLMs do not have sufficient accuracy for clinical settings. **T2:** There are difficulties with designing and validating a suitable prompting strategy for the P4. **T3:** LLMs are purely stochastic models and thus the P4 will not able to understand or critically engage with the information it is being trained on. **T4:** The P4 relies on logically invalid inductive inferences by analogy and therefore will never be able to predict with complete certainty what the patient’s preferences would be.

We now elaborate each concern and offer our response.

### T1: LLMs Do Not have Sufficient Accuracy for Clinical Settings

Several critics doubted whether the current technical capabilities of LLMs are sufficient to fulfil the proposed aims of the P4. For example, Biller-Andorno et al. [28] suggest that “while zero-shot performances of LLMs are impressive, their accuracy is not sufficient in clinical settings” (p. 36). They highlight that “the lack of robustness of LLMs is a problem in medical applications” [28], and express concern about the fine-tuning process. Similarly, Starke and Jox [31] point out that “LLMs have been shown, at least sometimes, to provide inconsistent outputs, to take mutually exclusive stances, and to lead to morally problematic judgments based on their sensitivity to framing” (p. 43). They note that it “seems overly optimistic that a P4 could in the near future overcome these fundamental [technical] challenges” [31]. Finally, Soffer et al. [32] highlight the “subtle blind spots in complex reasoning tasks” that exist in current LLMs, and how this may limit “the models’ ability to navigate nuanced ethical situations.”

We agree that building a P4 poses significant technical challenges. However, we see no in-principle reason to think these challenges are insurmountable, particularly in light of the recent preliminary success of the aforementioned P4-like prototype (developed, ironically enough, by Starke and Jox themselves) [14]. In our original paper [13], we argued that there is evidence that LLMs are capable of inferring non-medical preferences to a high degree of accuracy and therefore, by analogy, we assume that they are likewise capable of inferring medical preferences, if trained on the right kind of data (more on this below). Research in this area is progressing at a very fast pace. For example, in addition to the recent *NEJM AI* paper, a different study using interview data to personalize an LLM’s output found that the model could predict a wide range of preferences, attitudes, and values at an accuracy – about 85% – that approaches human test–retest reliability after two weeks [43]. Recent technical advances, for example in the use of chain-of-thought prompting by ‘reasoning’ models [44], as well as an accelerating pace of improvements on complex tasks more generally [45], suggest we have not yet reached the ceiling of possible accuracy. While the stakes may often be higher in medical settings, it is not unreasonable to assume that the ability of LLMs to infer values or predict responses/preferences in these other contexts would transfer, pending appropriate translational research, over to the medical domain. That being said, we accept that patterns of confabulation and error show that these technologies are sensitive to prompting [46], and that significant further work may be required to determine the optimal prompting strategy to increase both accuracy and reliability. We turn to this issue in the following section.

Ultimately, such issues will not be solved by argument but must be tested by experiment. We agree with Meier [47] who wrote: “let’s build them and find out” (p. 50). In sum, while we concede that there are numerous technical difficulties confronting the P4 proposal, and that LLMs currently do show many of the weaknesses identified by the commentators—for example, susceptibility to framing effects—we are not prepared to conclude that these difficulties cannot be overcome; moreover, humans also have some such limitations [48]. How the limitations of a P4, following technical improvements, will stack up against those of humans in relation to such criteria as accuracy and reliability is something that will have to be empirically determined.

### T2: Difficulties with Designing and Validating a Suitable Prompting Strategy for the P4

Some critics worry about the difficulties with designing and validating a suitable prompting strategy—partly for reasons touched in in the previous section. Sharadin [30], for example, highlights how “apparently content-irrelevant features of prompts (politeness, sophistication, word choice) regularly have substantial effects on model performance” (p. 64). This makes it hard to design a prompting strategy “suitable for eliciting a model’s actual prediction regarding an individual’s patient preferences” [30]. More generally, as Biller-Andorno et al. [28] point out, “there is still no consensus on the procedures to evaluate the performance of these models” (p. 36).

We agree that effective prompting will be key to the success of any LLM-based P4 system. Research in this area is ongoing, but advanced techniques (such as prompt tuning, prefix-tuning, and prompt “ensembling”) already show promise for mitigating unintended biases or artifacts introduced by specific prompt wordings and/or arbitrary variations thereof [49, 50]. Nevertheless, just as traditional software systems are susceptible to edge cases and unexpected inputs, and just as humans are likewise susceptible to, e.g., framing effects, AI systems may indeed exhibit varying behaviours based on the specific input or prompting. The sensitivity of LLMs to the way inputs are phrased is an important issue which will necessitate systematic testing of candidate prompt strategies alongside training protocols for human prompters.^5^

Suppose that such testing reveals an effective prompt strategy for stabilizing a P4’s predictions. There would still be the problem of *validating* these predictions, as various critics have noted (e.g., Biller-Andorno and colleagues, as mentioned above). We agree this is an issue; however, it is one that is not unique to the P4 proposal. Indeed, the validity of *any* method for predicting treatment-relevant preferences held by an incapacitated person when they were last autonomous will be hard, if not impossible, to demonstrate to the satisfaction of all. This is for several reasons:

1. We may have no specific record of what they wanted or how they thought about the issue before losing capacity.
2. We cannot confirm the answer with the person who now lacks capacity (although we should certainly inquire into their current attitudes and preferences insofar as they are capable of expressing these, as they may serve as clues to their previous, capacitated preferences and/or be worthy of consideration in their own right) [51, 52].
3. If treatment is withheld and they die, or otherwise never regain capacity, we cannot confirm with them, after the fact, if the decision was in accordance with their preferences (as held or endorsed when last autonomous). And:
4. Even if they are treated and do regain decision-making capacity, we cannot be sure that whatever they tell us at that point is the same as what they would have said before losing capacity – or even, in some cases, that they are the same person as the one who existed prior to losing capacity (e.g., if they have undergone certain so-called transformative experiences in relation to their illness or its treatment) [53–56].

Faced with these problems, we should be clear that the epistemic difficulty is inherent to decisional incapacity and cannot be eliminated by choosing a different decision standard, such as substituted judgment versus best interests, or by appealing to any particular tool or person. When the patient cannot be asked, and when there is no contemporaneous record that determinately settles the relevant question, there is no principled way to know *for sure* whether a given decision aligns with what this person would have wanted. The practical upshot, however, is not that we should abandon efforts at preference-sensitive decision-making, but that we should pursue the best available approximations and evaluate candidate methods comparatively.

One established approach, used in prior work on surrogates and the P3, is to present competent individuals with carefully specified hypothetical scenarios and compare their stated preferences with predictions generated by human surrogates, a P4, or other proposed predictors [9]. Where possible, this can be supplemented by studying individuals known to be, or retrospectively found to have been, close to losing decisional capacity, comparing predictions based only on pre-incapacity data to preferences elicited shortly beforehand and, where relevant, after recovery. These approaches do not resolve the underlying epistemic problem, but they represent the strongest available alternatives to abandoning preference-sensitive decision prediction altogether.

By using this methodology, we can ensure a fair comparison of the P4's accuracy to existing surrogate decision-making methods (see, e.g., Starke et al.’s [14] recent proof-of-concept in *NEJM AI* for an example of this). Further empirical testing would be necessary to determine what *level of accuracy* would suffice for patients and relatives to be willing to use the P4 technology (we present findings on this question in forthcoming work); and further ethical analysis will be needed to determine what level of accuracy would be needed to normatively justify the resources required to build and/or scale up the use of a P4 in practice; see Sect. 4.1.

### T3: LLMs are Purely Stochastic Models and thus the P4 Will Not Able to Understand or Critically Engage with the Information it is Being Trained on

Starke and Jox [31] argued that a P4 would not be able to understand and apply the “personal reasoning underlying a patient’s preferences” which they take to be necessary for respecting that patient’s (former) autonomy and which is “made possible by rich relational and narrative experience of the person” in question (p. 44) [31]. Similarly, Ferrario and Biller-Andorno [24], caution against “being overly captivated by the seemingly impressive performance of stochastic computations,” positing that “holding a comprehensive body of knowledge is not sufficient in itself for understanding the facts within” (p. 654).

It is unclear whether a P4 would be able to “understand” the personal reasoning behind a patient’s preferences (even assuming that the relevant preferences were formed through a process of reasoning, which may not always be the case). It depends on what it means to “understand” something and whether P4s, like other LLM-based systems, are capable of understanding in that sense. According to Pepperell [57], with some exceptions, recent theorists have tended to argue that “it is not a requirement that computer-based systems are capable of consciousness or genuine semantic appreciation in order to understand” (for example) a process of reasoning, and that they can in fact exhibit “understanding” in some relevant sense (p. 2). On the other hand, if “understand” is interpreted as requiring “seeing” or subjectively “grasping” something (perhaps accompanied by an *ah-hah* feeling of recognition as to how certain things “fit together”) [58]; then P4s, like other LLM-based systems, are unlikely to be capable of understanding in this sense, insofar as they are generally thought to lack subjective consciousness or *qualia* [59–61].

However, we are sceptical that understanding, in either sense, is necessary for the P4 to represent an improvement over the present state of affairs in surrogate decision-making. We see the P4's role as being relatively narrow—as a tool to provide information about relevant preferences or values (i.e., treatment-relevant preferences or values most likely held or endorsed by currently incapacitated patients when they were last autonomous; assume this qualification for “preferences” in what follows). It is also unclear why understanding the reasoning behind such preferences should be a primary goal of substituted decision-making. In order to accurately identify someone’s preferences, it may sometimes help to understand the reasoning behind them. But even if one *doesn’t* understand exactly why Grandpa, say, wants to have treatment either given or withheld under certain conditions (for example, if he is ever in a coma and has less than a 25% chance of recovering his full cognitive and emotional capacities), it is still helpful to know *whether* he wants treatment under those conditions if one is concerned about respecting his autonomy.

But suppose an understanding of the reasoning behind someone’s preferences *is* somehow indispensable to making substituted judgments. While it may be the case that there are some instances in which “rich relational and narrative experience of the person” will enable a surrogate to have access to the reasoning process(es) behind the preferences of a formerly competent patient, and, on that basis, to more accurately predict what they would have wanted in the current conditions, the generally low levels of accuracy exhibited by human surrogates when dealing with hypothetical scenarios might suggest this is not very common [5, 62, 63].

Again, much of the original motivation for both the P3 and the P4 is precisely this inability of human surrogates to consistently make accurate predictions about what their loved one would want under various conditions (much less establish the personal reasoning underlying those preferences).

Finally, even if one grants that a P4 could in no sense “understand” the underlying reasons for a patient’s preferences; and even if one further grants that an understanding of those reasons is important for surrogate decision-making, it might still be the case that a P4 could be helpful toward this goal. This is because a P4, like other LLM-based systems, *can* generate statements that take the form of reasons or justifications for its predictions or other outputs [64], and these reasons-statements can be critically evaluated by humans for their cogency and relevance [65]. For example,

a P4 might offer the following reason for why a patient would not wish to be kept alive when unable to communicate with family: “On June 6, 1990, John wrote an email to Jack expressing his sadness that Jack’s father could not communicate after a stroke, and said he would never want to be kept alive in a state like that.” That is a relevant reason. It is not decisive, but it ought to be evaluated in the context of all that is known about John [65] (p. 106-7).

Another concern raised by Starke and Jox [31]—that is, apart from the one having to do with LLMs’ purported inability to understand reasons—is that “a preference predictor in the shape of an LLM could, even after fine-tuning, fall back to replies supported by the pre-trained LLM, reflecting the view most frequently present in the training data instead of mirroring a patient’s potentially opposite views” (p. 43).

We see this as another addressable technical problem rather than a fundamental ethical critique. As a reminder, fine-tuning involves taking an existing model and training it on a further, more specialized training set. There are several technical choices involved in fine-tuning (e.g., how many layers are updated and for how many epochs), which in turn determine how strongly the model reflects its base training data versus the fine-tuning data [66, 67]. While it is true that some fine-tuning methods might privilege the former, others might privilege the latter. In the absence of empirical testing, the extent to which, or conditions under which, an eventual implementation of the P4 concept would favor one sort of data over the other can only be a matter of speculation. We’ll quote Meier [47] again: “let’s build them and find out” (p. 50).

### T4: The P4 Relies on Logically Invalid Inductive Inferences by Analogy and Therefore will Never be Able to Predict with Complete Certainty What the Patient’s Preferences Would be

Rzepinski et al. [29] contrast the P4 approach with examples of what they consider to be truly ‘personalized’ medicine, like gene therapy medicinal products (GTMP). They argue that while GTMPs rely on causal knowledge about molecular mechanisms, allowing for accurate predictions and personalization, the P4 operates instead on inference by analogy, an inductive process they deem logically invalid and insufficient for modelling an individual's specific preferences. Such modelling, they argue, demands a degree of certainty that inductive reasoning cannot deliver: "When making decisions based on the patient's personality profile reconstructed by P4, we want to be sure that the decision will not be characterized only by statistically satisfactory accuracy, but that it will *certainly* be consistent with the patient's preferences" (p. 51, emphasis added) [29].

We think this sets an unrealistically high bar. Few decisions made in medicine are based on perfect causal certainty or formal logical syllogisms. Instead, decisions typically rely on a combination of knowledge, available evidence, prior experience, intuition, and collaboration by clinicians [68]. As London [69] argues, while treatments are frequently implemented by clinicians with incomplete causal knowledge systems, it is often more important to know that something *does* work rather than necessarily knowing *why* it works.

Moreover, many successful applications of machine learning in high-stakes domains like medical diagnosis operate without explicit causal models yet still provide highly accurate and reliable predictions by capturing the complex correlational structure of data [70–72].

The P4, while not based on explicit causal models, has the potential to capture and leverage complex statistical patterns governing human preferences and decision-making processes. Researchers are currently attempting to develop causal reasoning engines to supplement LLMs and increase explainability [73, 74] — advances that could theoretically be integrated into the P4 as well. While we agree that it would be desirable to have certainty about the consistency of a P4’s predictions with patients’ preferences, we must reiterate that the P4 – like the P3 – is motivated by an *existing state of affairs* in which there is *very little certainty* to be had about the preferences of incapacitated patients.

No existing, or even proposed, approach to patient preference prediction offers anything like certainty grounded in causal models. We should avoid imposing success criteria on P4 or related technical solutions that no human surrogate—or even the patient themselves—could meet. Although it remains unclear what level of certainty or accuracy would justify developing or using a P4 (of course, the case would be strongest if a P4 were shown to *substantially* outperform human surrogates, as we discuss below), requiring 100% certainty is too demanding.^6^

Finally, our proposal does not frame the P4 as a standalone solution. Rather, like the P3 [9], it would in most contexts be used in conjunction with input from human surrogates, healthcare providers, and other domain experts who can provide additional context, validation, and reasoning. An imperfect P4 system could still represent a significant improvement over the status quo by providing additional relevant information to inform decisions.

## Data-related Concerns (D)

Several objections have been made regarding data extraction and sourcing process, which can be subcategorised as follows: **D1:** Privacy and confidentiality. **D2:** Authenticity and social media data. **D3:** Insufficiency of mere preference data. We tackle each issue in turn.

### D1: Privacy and Confidentiality

Berger [34] raises concerns about privacy and confidentiality, asking “who ought to have access to texts, personal emails, and other non-public facing correspondence in order to then allow AI to use it in its ‘learning’ about the patient?” and, “does the motivation to serve the patient through the rendering of more highly individualized decisions justify plying the depths of the patient’s digital self?” [34] (p. 28).

These are important questions. One answer is that, ideally, individuals would prospectively and voluntarily grant permission to access the relevant data sources before losing capacity. As we wrote in our original paper, “the patient’s rights to privacy include the right to share data if and as they want, so they can consent to this use of their data” [13] (p. 7). We also outlined several options for cases in which consent could not be obtained before capacity was lost. These include relying on proxy consent from a human surrogate or limiting inputs to publicly available information (such as blog posts or social media activity). Although this approach may be less accurate, it could still provide useful guidance for surrogates.

It is also important to note that there is no technical requirement for personal data to be shared with third parties. Several powerful open-source LLMs can be adapted to run on local systems. A P4 deployed locally would not require individual-specific—or indeed any—data to be transmitted externally. Privacy and confidentiality concerns can therefore be mitigated through technical design choices, including the use of locally run, open-source models.

### D2: Authenticity and Social Media Data

Another major criticism concerns the proposed use of social media activity as training data, with particular concerns raised about the authenticity of predictions derived from such data. Rahimzadeh [39] observes that “many users often post on social media only the version of their life they want others to see” (p. 30). Similarly, Ballantyne and Style [26] note that social media posts are “typically designed to present a curated view of the person” (p. 56), while Berger [34] emphasizes that the “relationship between one’s social media use and one’s social identity is highly complex” (p. 27). As Gligorov and Randall [36] argue, “publicly expressed preferences of patients, made outside the context of end-of-life decision-making, are often mediated by many considerations, such as fear of reprimand and judgment” (p. 54).^7^

These objections converge on the idea that social media posts (among other types of data believed to be largely “unrelated” to medical preferences) may distort predictions by reflecting “social norms and contingent factors” rather than an individual’s core beliefs and values [38] (p. 41). And no doubt, people’s behavior on social media often does likely fail to reflect their authentic self, on a range of ways that term can be understood. That is why we proposed various *other* sources of data (such as structured interviews, questionnaires, electronic health records, and so on) that could be used in addition to, or instead of, social media or other “public-facing” data.

Nevertheless, we will say a few words about the potential use of social media data for purposes of training a P4. First, there is evidence that online environments can, in some cases, *facilitate* the expression of a person’s so-called “true self” [76], especially for those who struggle with face-to-face disclosure [77]. What is more, “those who feel more able to express their ‘true self’ online” (based on a psychometric scale developed by McKenna et al. [78]) appear to leave empirically detectable “signatures,” such as posting more frequently on Facebook, posting “more personally revealing and emotional content, even controlling for how much time they spend on the site,” and posting more frequently on others’ walls [79] (p. 371).

This suggests that it *might* be possible to distinguish between users whose social media activity is more, versus less, expressive of their authentic selves based on additional types of data (regarding frequency of posting, and so on) that is also available online.^8^ At least, it is a possibility that should be explored.

But now let’s suppose we accept the “authenticity” critique. It is not unique to social media-derived data. While there may be some disanalogies—for example, in certain procedural elements by which the relevant data are collected—traditional advance care planning can *also* trigger inauthentic self-expressions. People can feel pressure to present polished or socially desirable versions of themselves: for instance, emphasizing family-oriented or religious values while deemphasizing values in tension with these, such as those around personal freedom or self-fulfilment.

Thus, the challenge of authentic self-representation in healthcare planning extends beyond digital platforms. In fact, it may be an inherent limitation of any prospective decision-making process in which one’s stated or inferred preferences may be disclosed to – or otherwise known by – others on whom one would like to make a certain impression. We are therefore sceptical it is a specific shortcoming of the P4 and (some of) its potential data sources. Even so, we must reiterate what was stated in our initial proposal—that wherever possible, individuals should prospectively indicate which types of information they would want used, and that preferences against the use of social media data should be respected. Where, by contrast, it is not possible to obtain prior consent, such that surrogates must decide whether data of various types should be used, they must be bound by all relevant ethical and legal standards regarding proxy data authorisation.

### D3: Insufficiency of Mere Preference Data

Some have argued that relying on preference data alone will be insufficient for accurately determining what patients would truly want in medical situations. Gligorov and Randall [36], for instance, argue that effective prediction requires sophisticated theory of mind capabilities that can accurately reconstruct not just individual beliefs, desires, and fears within medical contexts, but also the complex interrelationships between these psychological elements. Tretter and Samhammer [33] argue that AI-based preference predictors like P4 cannot adequately capture the full spectrum of human personalities and diverse range of desires and goals that are essential for effective shared decision-making (p. 175). And Cordeiro and Kirjanenko [16] emphasize the value of maintaining human surrogates in the process, as they can sometimes provide superior insights into patient values and preferences due to their intimate knowledge of the individual (p. 460).

Meanwhile, Herington and Kluger [37] observe that patients themselves often struggle to accurately predict their future preferences, and that preferences expressed outside structured advance care planning processes tend to be unreliable (p. 32). This unreliability is compounded by the gap between what people state explicitly and how they otherwise communicate their values. Rzepinksi et al. [29] challenge the assumption that adequate preference modelling can rely solely on verbalized data, describing this approach as an oversimplification that ignores the diverse non-verbal channels through which preferences are conveyed, including hand gestures and facial expressions (p. 52). Finally, Bishop [35] contends that P4 systems operate through static demographic and patient data, creating linear projections that cannot accommodate *transformative* elements of human experience such as spiritual insights, existential revelations, or the influence of familial love—elements that resist formalization but remain central to authentic human decision-making (p. 46).

These critiques rightly highlight the complexity of human decision-making and the limits of relying on verbal or written data alone. However, we worry that they may overestimate what human surrogates can achieve while underestimating the potential of the P4. Moreover, using a P4 need not exclude other tools, information sources, or human decision-makers; indeed, we have argued that it ideally should *not* be used in isolation [21].

We must again recall the status quo. Available evidence suggests that human surrogates, despite their more or less intimate knowledge of patients, often fail to accurately predict their loved one’s preferences [5]. In response to Bishop’s concern about the “static memory” of LLMs, we argue that this critique seems to assume that human memory and decision-making processes are somehow more dynamic or reliable. However, human surrogates also rely on their own “static memories”^9^ of past conversations, observed behaviours, and known preferences when making decisions on behalf of incapacitated patients. One key difference is that the P4 can potentially access and analyse a much broader and more comprehensive dataset than any individual human surrogate could retain or process.

Additionally, individuals can lack available surrogates altogether [75]. Against this backdrop, even an imperfect P4 could represent a substantial improvement—notwithstanding the sorts of limitations to predictive accuracy raised by our critics. Indeed, there is evidence suggesting that a P3, using only basic demographic data, could be *just as* accurate as human surrogates in predicting an individual’s preferences [5, 8]; and the aforementioned *NEJM AI* prototype of a machine learning-based P4 analog reported accuracy that matches or *exceeds* typical estimates of human performance [14]. Therefore, it seems reasonable to hypothesize that a personalized model, i.e., one that is fine-tuned on data *specific* to the individual, will only increase this accuracy, possibly by a significant margin.

In addition, while the initial prediction by the P4 may be based purely on inputted textual data, these predictions can be refined through interactions with human surrogates (if available and appropriate) and healthcare professionals. Further, current state-of-the-art LLMs are multimodal; as a technical matter, it is possible to use video, sound, and other modalities in addition to text for fine-tuning purposes. We thank Rzepinski et al. [29] for this important insight.

Regarding the assertion of Herington and Kluger [37] that “patients themselves are often poor predictors of their future preferences” (p. 32) and that even preferences expressed in the context of traditional advance care planning processes may be “unreliable,” we can only reiterate that the P4 must be compared against what is possible given the status quo. Of course, one could take an extreme position and argue that the preferences captured by traditional processes should be disregarded due to their potential unreliability (e.g., that advance directives should be considered invalid due to individuals’ being poor predictors of their own future preferences); however, that would take us off in another direction.

## Justification for Required Resources Over Alternatives (J)

Several authors have argued that in order to justify the resources required to design and implement a P4 in clinical settings, as well as any potential ethical harms associated with its use, the P4 would need to be *significantly* more accurate than current surrogate standards, rather than needing to simply be more accurate by some margin or another [26, 28, 38, 39]. Along these lines, Mertes [38] also argues that increasing the P4’s predictive power would require efforts and energy that exceed its value over advance care directives.

These concerns break down into two main issues: **J1:** Required accuracy of P4 to justify associated harms. **J2:** Limited resources should prioritize improving advance care directives rather than developing the P4.

### J1: Required Accuracy of P4 to Justify Associated Harms

Several authors have critiqued our initial assessment that the P4 “would only have to be somewhat more accurate than [the current baseline accuracy of human surrogate decision-making] to be useful for present purposes” (p. 5) [13]. For example, Ballantyne and Style [26] suggest that “this standpoint seems to ignore the substantial costs of a P4 model in terms of data security and governance, regulation, individuals’ time and effort to create a digital psychological twin, clinical training and integration into health pathways” (p. 56). Similarly, Biller-Andorno et al. [28] argue that “if a P4 were to be used not only as a substitute for absent surrogates but also to question their judgements or resolve disputes among them, it should demonstrably exceed the performance of surrogates” (p. 36). Rahimzadeh [39] further draws attention to the “growing debate about where we should set the evidentiary thresholds for AI systems and how to select appropriate metrics to compare them to human systems” (p. 30).

We acknowledge that developing and implementing the P4 would require significant investment in terms of resources and effort, and the necessary level of predictive accuracy to justify such costs is not self-evident. However, even a P4 with accuracy comparable to current surrogate decision-making might offer advantages along *other* dimensions apart from sheer accuracy that should also be factored into the equation.

For instance, it could provide faster predictions and, perhaps in some cases, help to alleviate the sometimes significant emotional burden on surrogates by providing them with an additional source of information to guide decision-making that may make them feel the decision is not solely on their shoulders [6]. A P4 may also be more up-to-date than surrogates. For example, it could be trained to include individual-level data from the past weeks or months, when the patient may not have been in contact with surrogates. In contrast, advance directives used by surrogates may be many years old. Moreover, for individuals without appropriate surrogates, a P4 with surrogate-level accuracy could represent a potentially major improvement in their care by providing at least some personalized, preference-based input that would otherwise be entirely absent from their care decisions.

Nevertheless, we concede that the strongest case for the P4 lies in its potential to significantly enhance accuracy over, or in combination with, existing surrogate or P3-based decision-making. In evaluating this potential, we must keep in mind the scale of the problem that decisional incapacity currently poses in healthcare systems worldwide. The issue of medical decision-making for incapacitated patients deeply affects a large number of people. Even modest improvements over the status quo (on a case-by-case level) could yield substantial system-wide benefits that could, in principle, justify considerable investment.

It is also worth noting that the P4 would not necessarily require a standalone research initiative. Instead, it could emerge as a natural application of broader agent simulation technologies that are already under development for other purposes. Rather than viewing this as a separate program that needs its own justification, we can recognize that these underlying technologies will likely be developed *regardless* of our specific intentions. The P4 would then simply represent one potentially beneficial way to apply these technological advances to serve society's needs.

In the short time since our paper was published, research and development in the area of personalized LLMs has exploded. For example, as briefly alluded to in the introduction, scientists at Stanford and Google DeepMind have developed “a novel agent architecture that simulates the attitudes and behaviours of 1,052 real individuals—applying large language models to qualitative interviews about their lives, then measuring how well these agents replicate the attitudes and behaviours of the individuals that they represent” [43].

According to a preprint describing the findings, the simulated agents’ responses to items from the General Social Survey^10^ closely matched the original participants’ responses, achieving a level of agreement corresponding to about 85% of the test–retest consistency that participants themselves exhibited when answering the same survey again two weeks later. The simulated agents also performed comparably in predicting personality traits and outcomes in experimental replications [43]. In practice, the training to produce the simulated agents looks something like this:

Imagine sitting down with an AI model for a spoken two-hour interview. A friendly voice guides you through a conversation that ranges from your childhood, your formative memories, and your career to your thoughts on immigration policy. Not long after, a virtual replica of you is able to embody your values and preferences with stunning accuracy [43].

If we now imagine a somewhat extended interview focused on medical preferences and associated values, with questions carefully tailored to elicit maximally informative and relevant responses, we could end up with yet another version of the P4, one based on data from personal interviews by AIs. This ‘digital psychological twin’ of the patient – or AI Simulation of an Individual Mind (AI SIM)^11^ – could then be queried about what the patient would like to have happen in whatever specific circumstances have arisen. If something like 85% accuracy could be achieved, that would be much better than most human surrogates, as established through existing (if imperfect) validation methods.

Time and testing will tell. However, even if such accuracy is achieved, we must acknowledge that various potential harms and disadvantages of a P4 (e.g., susceptibility to data privacy concerns) would also have to be tallied up, while anticipating the various different types of questions and clinical circumstances that might arise in surrogate decision-making. We consider these issues a priority for future work.

Finally, although establishing a P4 system would require substantial upfront resources, the marginal cost per use could be relatively low once in place. This long-term value is important when assessing viability and should be weighed against the significant financial and emotional burdens of current surrogate decision-making. These include prolonged hospital stays, unnecessary or missed treatments resulting from judgment errors, and the psychological toll on families. If a P4 could meaningfully improve outcomes in even some of these areas, its benefits may outweigh its financial and other costs (such as environmental impacts). That said, we would welcome a formal cost–benefit analysis to more rigorously evaluate under what conditions a P4 is likely to be net beneficial or net harmful.

### J2: These Resources would be Better Allocated to Improving Advance Care Directives

Critics have also questioned whether investing in the P4 is preferable to strengthening existing advance care planning. Mertes [38], for example, argues that it is “difficult to justify why one should invest in the detour through the P4 system rather than in advance directives to find out what the incapacitated patients prefer” (p. 42), comparing the latter to a “simple hammer that would have done the trick much more efficiently” (ibid.). Similarly, Gutiérrez-Lafrentz et al. [40] argue that “anticipatory dialogues are an irreplaceable human process that should not be given up” and that the P4 should “foster ACP [Advance Care Planning] rather than replace it” (p. 58).

In an ideal world, every individual would have a recently updated advance directive that directly and correctly addresses the specific situation in which they might find themselves incapacitated—one that is found, used, and rightly interpreted by the physician. This approach, while seemingly obvious and simple, has been pursued for decades with limited success. Indeed, the original P3 papers by Rid and Wendler [8, 9] were motivated by this very state of affairs. They reviewed efforts up to that point to improve rates of uptake and found them severely lacking. Regrettably, the situation has not significantly improved since then [82].

What is more, a recent summary of the literature by Morrison et al. [83] reports that even where ACP is in place, it fails on several accounts to provide the hypothesized benefits. The review found no evidence that ACP influences medical decision-making at the end of life, enhances the likelihood of goal-concordant care, or improves patients' or families' perceptions of the quality of care received. Additionally, there was no association between ACP and subsequent health care use, including emergency department visits, hospitalizations, and critical care.

Given this context, namely decades of sustained efforts to promote advance care planning with limited uptake and mixed evidence of downstream benefits even when ACP is in place, we think it is reasonable to explore complementary approaches rather than relying on further incremental improvements alone. In this light, the development of a P4 could be a valuable addition to existing practices.

Moreover, unlike rather static advance directives that can quickly become outdated, the P4 aims to infer an individual's underlying preference structure through the analysis of their personal data. This adaptable approach, we argue, may allow the model to respond dynamically to the specific situation the patient finds themselves in—using the most current information available—rather than being constrained by the limited information provided in an advance directive or via an ACP process. Additionally, as mentioned previously, a P4 could be used to ‘auto-fill’ advance directives [26] (Box 1), thereby actually increasing participation in advance care planning, and improving the quality of advance directives.

## Significance of Human Involvement and Potential Impact on Relationships (R)

One of the central themes in the various commentaries is the concern that the P4 poses a threat to human relationships, as follows. **R1:** Harm to the doctor-patient relationship. **R2:** Harm to family relationships. While some of us have sought to address these concerns in a separate paper [22], we will briefly summarize the main objections here and our responses.

### R1: Harm to the Doctor-patient Relationship

There is a perceived risk that the P4 system could undermine the doctor-patient relationship. Unsurprisingly, then, a recent study by Benzinger et al. [84] that assessed the attitudes of German anaesthesiologists and internists towards the use of AI-driven preference prediction tools revealed an overall hesitance amongst physicians to use this kind of technology. The study found that physicians were particularly concerned about the lack of explainability in AI decision-making and the potential loss of individuality in patient care, with many expressing worries that AI could not adequately account for the nuanced contextual factors that characterize ethical deliberation.

However, it is worth noting that the same physicians acknowledged potential benefits of AI systems, including increased objectivity, reduced bias, and time-savings in ethical support—advantages that may ultimately enhance rather than diminish the quality of patient care. Further, these concerns about de-humanization may reflect unfamiliarity with AI technology rather than inherent limitations of the P4 system. As a reminder, we envision the P4 functioning as a supplement to, rather than a replacement for, human judgment in ethical decision-making.

From a patient perspective, one of Mertes’ expressed concerns is that patients may feel uncomfortable at the prospect of their conversations with their clinician being recorded, or that this will impede on the candidness and openness of the conversation (p. 42) [38]. However, these conversations would only be recorded after a detailed discussion with the patient regarding its purpose, the possible benefits and harms, and implications of this data collection method. Explicit consent would be obtained before proceeding with the conversation. Failing this, there still exist several other data collection methods that the patient may feel more comfortable using.

Finally, Riddle [41] worried about the P4 resulting in the removal of the clinician from the decision-making equation altogether. According to Riddle, if the P4 is to be useful as a largely automated process – i.e., one that might be less prone to making certain types of error to which humans are prone, such as panicking in a stressful situation and reasoning poorly – clinicians would then need to be effectively “removed” from the decision-making process. But, Riddle thinks, this would have other harmful effects, since clinicians have distinct abilities and contributions to make.

However, we believe this is a false dichotomy: “*either* clinicians are ‘removed’ from the decision-making process, with the P4 (so to speak) ‘running the show,’ *or* clinicians are appropriately involved, and the P4 loses its value” [22]. Although it possible that any necessary “clinician involvement might come at the cost of *some* of the potential benefits a fully automated system could provide … it could still be the case that the adjunctive use of a P4 would provide *enough* value added to be worth pursuing” [22]. The key is to achieve an appropriate equilibrium between human involvement and P4 automation. This presents a significant challenge that will undoubtedly require careful consideration, but we don’t believe it is in principle insurmountable.^12^

### R2: Harm to Family Relationships

Regarding family relationships, the main concerns that have been raised are that the P4 would undermine the collaborative nature and mutual understanding that is paramount for shared decision-making [37], and that surrogates may struggle to advocate for their own views when presented with a contradictory prediction from the P4, perhaps feeling that they now have a greater “burden of proof” to meet in advancing their own perspective [39].

Blumenthal-Barby et al. [23] worry that since “the P4 purports to deliver a quantitative and direct ‘answer’ to family member and clinical teams struggling with a morally and emotionally complex choice,” it may be psychologically difficult to resist the feeling that one should go along with that answer (p. 421). Cordeiro and Kirjanenko [16] also argue that relying on P4 output while excluding close relationships from decision-making processes could strengthen paternalistic tendencies among healthcare providers. They argue that the decision-making conversation should be broadened to encompass family members, close friends, and support workers rather than limiting it to physicians and AI systems.

These are valid concerns, and each is addressed at length in a separate publication [22]. But let us make a few observations here. Firstly, we envision the use of the P4 being optional. Unless it is known that the patient wanted it to be used, in which case that should be the presumptive option, families should provide input into how (or if) they would like to engage with the algorithm’s outputs.

Secondly, with respect to the “burden of proof” [39] that surrogates may have to offer in the event of differing predictions from the P4, this expectation already exists with current protocols. Conflicts of opinions between clinicians and families are already rife [85], and there exists an expectation that both sides are able to provide reasonable evidence behind their suggestions. If families wish to bypass the scenario where they may have to justify their reasoning against a potential differing suggestion by the P4, they may elect not to consider its output at all.

Alternatively, if families do proceed to engage with the P4, they should receive appropriate counselling on how it would work and how its output would be used (i.e., as another source of evidence to consider). As discussed in our separate paper [22], it would also be paramount to implement appropriate institutional polices that would ensure human surrogates (both the medical care team and family/other relevant human surrogates) ultimately remain responsible for treatment decisions.

Another possible benefit of the P4 process is that it could help facilitate important discussions, especially when surrogates advocate for decisions that differ from the P4’s prediction, potentially revealing whether surrogates are truly acting in accordance with substituted judgement principles. For example, these discrepancies could encourage surrogates to articulate their reasoning and potentially examine whether other factors—such as their own emotional needs, financial considerations, or personal preferences or values—may be influencing their advocacy. This process could ultimately strengthen the quality of surrogate decision-making by making underlying motivations more transparent and ensuring that treatment decisions remain genuinely patient-centred. We acknowledge, however, that this potential conflict could also be a source of stress for surrogates. Careful empirical testing of the P4 is needed to evaluate this possibility.

Beyond this potential benefit, the P4 may also help address challenges that arise in the absence of clear guidance. Acting as a legal representative can be especially difficult when long-standing family roles and authority structures are reversed [86]. For example, an adult child who has always deferred to an authoritarian parent may struggle to assume decision-making authority once that parent loses capacity. In such cases, a P4 could help support the child in navigating this role reversal and accepting their new responsibilities.

## A Final, Theoretical Concern

Before closing, we want to respond to Schwan [42], who raised a fundamental theoretical question about what the P4 should aim to predict: the choice that the individual themself would make if capacitated, or the choice that is most consistent with the individual's values and fundamental commitments.

We agree with Schwan that the latter approach is more appropriate. The goal of the P4 is not to predict whatever specific decision the person would in fact make—including those potentially driven by irrational fears, weakness of will, or temporary emotional states—but rather to identify treatments consistent with their deeper preferences and values. For instance, we should not reject IV medications simply because the patient had a morbid fear of needles that prevented them from consenting when capacitated.^13^ This clarification is crucial for understanding substituted judgment: we aim to increase the chances that patients are treated consistently with their authentic preferences and values, which in most cases aligns with what they would choose for themselves, but importantly diverges when psychological barriers or momentary lapses would lead to choices inconsistent with their fundamental commitments.

## Conclusion

We are grateful to all who have engaged with our original proposal for their valuable insights. Their contributions have highlighted important considerations and will help guide future development of the P4 concept.

Of course, ours is not the only proposed method for leveraging AI technologies to improve end-of-life care. In addition to the original P3 by Rid and Wendler, there are several other innovative approaches being explored. Meier et al. [88] have proposed a broader algorithm-based system for ethical decision-making in clinical settings. Biller-Andorno and colleagues [89] have developed the concept of an "Advance Care Compass" that aims to digitally transform advance directives by providing a structured, interactive framework for eliciting and contextualising patients’ goals and values to support advance care planning and shared decision-making. By contrast, the P4 is not primarily an elicitation or documentation tool, but a predictive decision-support system intended to approximate a patient’s likely preferences in situations where advance directives or contemporaneous input are unavailable or insufficient. More directly related to preference prediction, one of us (Sinnott-Armstrong) is working on an approach involving asking individual patients to make choices in samples of concrete scenarios [90]. As mentioned previously, there is also the possibility of using in-depth AI-based interviews [43].

We emphasize that developments in AI are progressing at a rapid pace. For example, a recent proof-of-concept study by Nolan et al. [25] showed that pre-trained LLMs can generate medically reasonable treatment recommendations from simulated clinical notes and patient value profiles. Even if, contrary to our argument, a P4 implemented with current technology were not yet more accurate than human surrogates, there is still good reason to discuss and develop the concept now. Given the pace of AI development, such systems are likely to become feasible—and potentially superior to existing approaches—in the near future.

At this juncture, we believe it is time to build and test a prototype P4, complementing the machine learning-based approach of Starke et al. (in *NEJM AI*) with the LLM-based approach we have proposed. Many of the key issues we now face are empirical in nature and can only be resolved through practical development and testing. Any implementation of the concept should be preceded by qualitative and ethical research in addition to technical testing. Initially, it should only be implemented as a decision-support system and only with explicit (proxy) consent for each type of data used.

## Data Availability

No datasets were generated or analysed during the current study.

## Footnotes

1 It has recently been argued that the substituted judgment standard does not, in fact, derive its normative force from respect for a person’s (former) autonomy [1]. However, this departs from the standard account [2] which we have defended elsewhere [3]. Although we acknowledge that respect for autonomy does not exhaust the set of morally relevant factors in surrogate decision-making [2, 3], it is nevertheless widely agreed, even in cultures that prize individual autonomy (relative to, say, family harmony) somewhat less than so-called Western ones [4], that at least *some* normative weight should be placed on the patient’s own preferences and values, as held when they last had capacity, when deciding what to do on their behalf.

2 E.g. [10, 11]; but see [12] for a response.

3 See also [15].

4 E.g., Blumenthal-Barby et. al 2024 [23]; Ferrario and Biller-Andorno 2024 [24]; Nolan et al. 2024 [25].

5 Of course, relying solely on human “training” protocols might not achieve the desired outcome of (sufficiently) more nuanced understanding and use of a P4 system. Thus, it will be important for designers of P4 systems to build in safeguards against foreseeable misuse or misinterpretation, such as by only making well-tested prompts available for selection, including frequent reminders of the P4’s limitations, and so on.

6 It also bears emphasizing that many individuals simply do not have a surrogate decision-maker [75], or at least one who knows them well and has their best interests at heart. For these individuals, it is difficult to envision a better alternative for approximating their former preferences than the P4 or something like it. At the same time, the use of any decision-support tool in the care of unrepresented patients raises distinct procedural justice concerns, given their heightened vulnerability. These concerns are not unique to the P4 and would need to be addressed through appropriate institutional safeguards, rather than by excluding such patients from potentially preference-sensitive tools altogether. We thank an anonymous reviewer for raising this point.

7 Others have raised similar concerns. Milian and Bhattacharyya [20], for instance, caution against incorporating “unrelated sources, such as social media posts, text messages and blogs, which are often misleading or entirely disconnected from genuine medical values” (p. 456), while Blumenthal-Barby et al. [23] warn that such materials may lead to “inaccurate assumptions about a person’s medical treatment preferences, and more broadly, their genuine desires, beliefs, and preferences” (p. 420).

8 See also [80] on how such data can be used to predict users’ personality traits, using data from the website formerly known as Twitter. As a reviewer notes, however, it will be important to see whether such findings replicate or remain consistent over time, especially as social media and the wider internet landscape continue to evolve.

9 Actually, human memory is essentially reconstructive and prone to change over time, whereas an LLM’s internal representations, once fixed, remain stable. This gives LLMs an advantage with respect to consistency, though not necessarily with respect to truth or understanding.

10 The General Social Survey is a detailed sociological survey designed by the National Opinion Research Center (NORC) to assess diverse attitudes, concerns, experiences, and behaviours of survey respondents. The survey takes about 90 min to complete, and is usually administered in person. In addition to demographic information, questions cover participants’ views on topics “ranging from government spending to the state of race relations to the existence and nature of God” (see: https://en.wikipedia.org/wiki/General_Social_Survey).

11 See Voinea et al., forthcoming [81].

12 An anonymous reviewer notes their respectful disagreement: “A fully optional, voluntary P4 might nevertheless exert pressure on surrogates to feel as though they are obligated to use it, which is compounded when staff distress on perceived deviations from patient preferences is taken into account. I don't have a good argument that this really is an insurmountable problem, but I think that the P4 is sort of a Pandora's box. Once it's implemented clinically, its influence could potentially become widespread and hard to critically quantify.” We agree this is a possible risk. To reduce it, we would advocate small-scale initial implementation in carefully controlled study environments, while collecting both qualitative and quantitative data on, e.g., perceived pressures to defer to the P4. In this way, researchers could build out the evidence base regarding likely outcomes under different real-world conditions prior to any “widespread” use.

13 See, e.g., Enoch 2017 [87] for a related discussion.

## Declarations

### Disclaimer

This research was supported in part by the Intramural Research Program of the National Institutes of Health (NIH, CL090123-01). The contributions of the NIH author(s) are considered Works of the United States Government. The findings and conclusions presented in this paper are those of the authors and do not necessarily reflect the views of the NIH or the U.S. Department of Health and Human Services.
